# Examination of out-of-hospital cardiac arrest patients with the Utstein style in Saga prefecture, Japan

**DOI:** 10.1186/cc10874

**Published:** 2012-03-20

**Authors:** T Iwamura, Y Sakamoto, N Kutsukata, T Hitomi, K Seki, M Koga, T Yamashita, A Nakashima, Y Nishimura, M Yahata, K Yamada

**Affiliations:** 1 Saga University, Saga, Japan

## Introduction

Saga Prefecture is a small prefecture with an area of 2,439 km^2 ^(place-of-residence 1,339 km^2^), a population of 849,709, and is located in northwestern Kyushu in the western part of Japan. Saga University has the only medical department in Saga Prefecture, Japan, and it is in charge of both the online and offline medical control of Saga. This report examined the present status of OHCA in Saga, which should be improved, and it aimed at exploring policies that can contribute to the improvement in a ROSC rate.

## Methods

The study examined 785 OHCA cases using the emergency conveyance record (the Utstein style) submitted for the purpose of MC verification by the fire-fighting organization in Saga from 1 July 2010 to 31 June 2011. The fire-fighting organization was classified into five areas (A to E) for every near medical classification. Comparative examinations were conducted between the background (age, gender, cardiac arrest cause, initial waveform, and hospital waveform, witness, bystander CPR, oral instruction, and pre-hospital medical examination (shock, advanced airway management, and drug use)) and the ROSC rate between the five areas. Statistical analyses included the chi-square test and Fisher's test.

## Results

Age, gender, cardiac arrest cause, initial waveform, witness, shock and drug use pre-hospital did not differ significantly between the five regions. The ROSC rate was significantly higher in A and C areas than in D and E areas (A: 40.1% to D: 24.4% *P *< 0.01, A: 40.1% to E: 26.8% *P *< 0.05, C: 39.9% to D: 24.4% *P *< 0.05), and the ROSC rate of a hospital waveform of asystole was significantly higher in A and C areas than in the other areas (A: 32.0% to B: 15.3%, D: 13.2%, E: 12.2% *P *< 0.01, C: 27.8% to B: 15.3%, E: 12.2% *P *< 0.05). There were significantly fewer examples of oral instruction enforcement in the E area in comparison to the other areas (E: 39.7% to A: 62.5%, B: 65.7%, C: 65.9%, D: 62.0% *P *< 0.01), and there were fewer examples of CPR enforcement in the D and E areas in comparison to the B and C areas (D: 50.8% to B: 63.9% *P *< 0.05, E: 42.3% to B: 63.9% *P *< 0.01, E: 42.3% to C: 59.7% *P *< 0.05). CPR was not always delivered without oral instruction because the bystander CPR-less rate of the oral-instruction-less example to citizens was not less than 80% in all the areas.

## Conclusion

An improvement of the quality of oral instruction could improve the ROSC rate. BLS education to the area, a re-examination of the oral instruction manual in the applicable areas, and the suitable evaluation of various examples of agonal respiration are together expected to improve the ROSC rate.

